# Tensile Property of ANSI 304 Stainless Steel Weldments Subjected to Cavitation Erosion Based on Treatment of Laser Shock Processing

**DOI:** 10.3390/ma11050805

**Published:** 2018-05-16

**Authors:** Lei Zhang, Yue-Hua Liu, Kai-Yu Luo, Yong-Kang Zhang, Yong Zhao, Jian-Yun Huang, Xu-Dong Wu, Chuang Zhou

**Affiliations:** 1 School of Food and Biological Engineering, Jiangsu University, Zhenjiang 212013, China; zhangleifd@ujs.edu.cn (L.Z.); fdjy2017@gmail.com (Y.-H.L.); zz87jy@yeah.net (X.-D.W.); callmezl@126.com (C.Z.); 2 School of Mechanical Engineering, Jiangsu University, Zhenjiang 212013, China; 3 School of Electro-Mechanical Engineering, Guangdong University of Technology, Guangzhou 51006, China; 4 AVIC Chengdu Aircraft Design & Research Institute, Chengdu 610041, China; zhao13683473316@sina.com (Y.Z.); 611@avic.com (J.-Y.H.)

**Keywords:** laser shock processing, tensile property, cavitation erosion, fracture morphology, laser weldment

## Abstract

Tensile property was one important index of mechanical properties of ANSI 304 stainless steel laser weldments subjected to cavitation erosion (CE). Laser shock processing (LSP) was utilized to strengthen the CE resistance, and the tensile property and fracture morphology were analyzed through three replicated experiment times. Results showed tensile process of treated weldments was composed of elastic deformation, plastic deformation, and fracture. The elastic limit, elastic modulus, elongation, area reduction, and ultimate tensile strength of tensile sample after CE were higher in view of LSP. In the fracture surface, the fiber zone, radiation zone and shear lip zone were generated, and those were more obvious through LSP. The number and size of pores in the fracture surface were smaller, and the fracture surface was smoother and more uniform. The dimples were elongated along the unified direction due to effects of LSP, and the elongated direction was in agreement with the crack propagation direction. Their distribution and shape were uniform with deeper depth. It could be reflected that the tensile property was improved by LSP and the CE resistance was also enhanced.

## 1. Introduction

Cavitation erosion (CE) usually appears in a flowing system, as the local pressure falls below the vapor pressure of the liquid. During this process, the oscillation behavior of cavitation bubbles is generated. These bubbles occur at the low pressure region and subsequently collapse when the pressure above the vapor pressure. As a result, pitting or erosion is generated on the material surface and the failure happens as CE destroys the mechanical properties of the materials [1]. At present, ANSI 304 stainless steel (ANSI 304 SS) is widely used in parts of water pumps, valves, and blades, due to its characteristics and cheap cost. However, CE can deteriorate the ANSI 304 SS components, including the welding parts, which threatens the material performance [2,3,4].

Nowadays, there are some approaches to mitigate CE. Firstly, the structure of some facilities was designed scientifically and reasonably to relieve CE, i.e., the transformation of valve [5] and the optimizing of pump impeller [6]. Secondly, selecting resistant material or applying a protective layer was adopted, which may be more convenient. Because CE is a surface phenomenon, the surface modification is a natural route employed in improving the CE resistance of facility components [7]. As is known that laser shock processing (LSP) is an advanced surface modification technology, the high strength impact waves can be induced due to the interaction between laser beams and the material surface. Because laser beams have high power density of GW/cm^2^ and short pulse of ns, the compressive residual stresses of several hundreds MPa are induced, thus the mechanical properties of materials can be improved [8,9].

Li et al. [10] found that CE resistance of the welding zone of nickel-aluminum bronze weldment was different from that of the heat-affected zone, because the microcracks causing cavitation damage initiated at the phase boundaries. This phenomenon also occurs in ANSI 304 SS laser weldments, although the mechanical properties of ANSI 304 SS processed by the fiber laser welding method were better than that of conventional methods [11]. Then, the electrochemical corrosion resistance of ANSI 304 SS laser weldments was studied subjected to CE, with the studies on the surface roughness, residual stress and morphology of cross section before and after LSP treatment [3]. In addition, micro-hardness, X-ray diffraction (XRD) spectra, and surface morphologies of ANSI 304 SS laser weldments were subsequently researched based on the effects of LSP [2,4]. As a result, surface roughness was reduced, micro-hardness was increased, and residual stress was introduced both in the laser welding zone (LWZ) and heat-affected zone (HAZ) [2]. It can be revealed that the CE resistance is improved to a certain extent for ANSI 304 SS laser weldments subjected to CE. 

Based on the above-mentioned research, it may be concluded that on the one hand, LSP enhances some indexes among the mechanical properties of the laser weldments and LSP is an efficient technology for the CE resistance. LSP will be utilized to strengthen the CE resistance of ANSI 304 SS laser weldments in this study. On the other hand, it is found that the tensile property is also the major index of mechanical properties [12,13,14], while it is not studied during CE in the previous work [2,3,4]. Chen et al. [15] found that the capability of the CE resistance could be represented by the tensile property. The tensile property plays an important role in the mechanical properties indeed, especially for the weldments. Xiong et al. [16] found that the comprehensive evaluation on the tensile strength and toughness and the effects of microstructure on the fracture of the weldments should be discussed. At the same time, high tensile strength is also demanded for the weldments subjected to CE. Hence, the tensile property of ANSI 304 SS laser weldments when suffering from CE will be supplemented in complementary research, and the process of the elastic deformation stage, plastic deformation stage, and fracture will be analyzed detail by detail. In addition, the materials may fracture due to the destructive effect of CE without any notice, thus the analysis of fracture morphology is also very important, because it represents the whole process of the fracture [14]. As a result, the reason and development process of fracture will be analyzed in detail, especially the three elements of fracture, including fiber zone, radiation zone, and shear lip zone in fracture surface, corresponds to crack initiation zone, crack propagation zone, and shear fracture zone.

In short, in order to systematically master the effects of LSP on the mechanical properties of the laser weldments subjected to CE, the tensile property of ANSI 304 SS laser weldments was treated by LSP, and the corresponding experimental data and tensile fracture were measured and analyzed in this study.

## 2. Materials and Methods

### 2.1. Materials and Laser Welding Procedure

**Figure 1:**
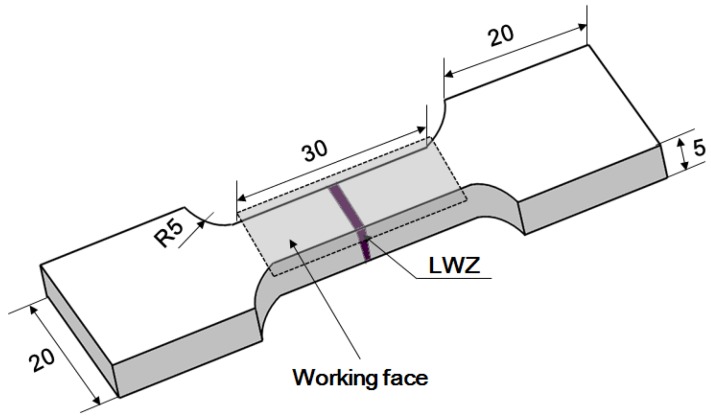
The dimension of the sample (unit: mm).

ANSI 304 SS sheets with a thickness of 5 mm were used as the welding material. The chemical composition of this material was: C (≤0.08), Si (≤1.0), Mn (≤2.0), Cr (18.0–20.0), Ni (8.0–10.0), S (≤0.03), and P (≤0.035). The sheets were treated using YLS-4000 fiber laser (IPG, Oxford, MA, USA). The butt weld was processed with full penetration. The laser welding parameters were: laser power of 4 kW, defocusing distance of −2 mm, and welding speed of 30 mm/s. The spot diameter of the laser beam was 0.27 mm with a focal length of 190 mm. Ultra-high mixed gas integrating helium (He) and argon (Ar) (1:1 mixing ratio) were used as the shielding gas during the laser welding procedure. The flow rate of adding gas was 0.6 m^3^/h in the condition of coaxial adding gas. The LWZ was located in the middle of the laser weldments and the width of the HAZ was small. Finally, the laser weldments were cut into the tensile samples, and they contained portions of the HAZ and LWZ in the center, as shown in Figure 1.

### 2.2. Experimental Procedure of Laser Shock Processing

The working face of the tensile samples was ground by SiC paper with different grades of roughness (from 150# to 1600#). Then, it was polished by the polishing machine with the revolving speed of 2000 r/min and metallographic polishing cloth was used cooperatively. Next, one group of the samples was processed by LSP. The working face of them was treated by a GAIA-R Q-switched Nd: YAG laser (THALES, Paris, France) with a wavelength of 1064 nm and the duration time of one pulse was about 15 ns (fixed parameter of equipment itself). The working face contained portions of the HAZ, LWZ, and some base metal in the center, as seen in Figure 1. The treated area of all the samples should be the same with the size of 30 × 10 mm. The repetition rate was 1 Hz, laser spot diameter was 3 mm, and overlapping rate was 50% between two adjacent spots to ensure no blind area at the LSP treated region. The laser pulse energy was 9 J. The 0.1 mm-thick sticky aluminum foil was compactly joined on the surface of the samples as an absorbing layer to improve the absorption from laser pulse energies and protect the material surface from laser ablation. A water layer with a thickness of 1–2 mm was used as the transparent confining layer to increase the peak pressure of laser shock waves, impulse in the samples, and action time [12,14]. Finally, the aluminum foil was torn off from the surface and the samples were cleaned in deionized water and degreased in ethanol by ultrasonic cleaning.

### 2.3. Cavitation Erosion Procedure of Tensile Samples

**Figure 2:**
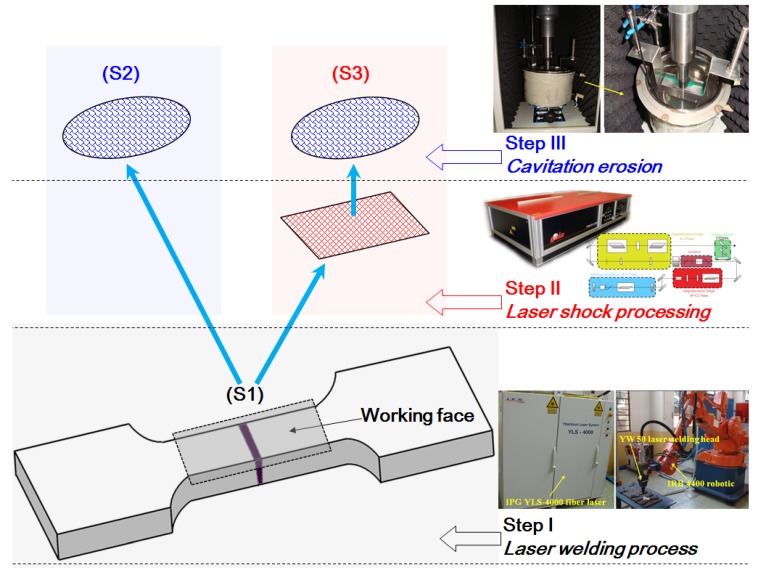
The process sequence graph.

The tensile samples with LSP impacts were slightly polished before CE test to avoid the interference of micro-indents by LSP, and the samples without and with LSP were subjected to CE. CE experiment was carried out in distilled water using an ultrasonic vibratory facility with a vibrating horn, according to ASTM G32-09 standard [17]. The testing parameters were presented in Table 1. The tip of the vibrating horn was submerged in water and aimed precisely at the middle of the samples containing the LWZ and HAZ. According to instructions of this facility, the best distance between the samples and the vibrating horn tip was 10 mm. The CE test lasted for 6 h. The process sequence graph of the above-mentioned experimental procedures was given in Figure 2. The samples were divided into three groups, i.e., original tensile samples without LSP and CE testing corresponded to S1, tensile samples without LSP after CE corresponded to S2, tensile samples with LSP after CE corresponded to S3. In addition, three samples were used in each group to ensure the validity of the experiments.

### 2.4. Mass Loss Measurement

The samples in S2 and S3 after CE testing and S1 were degreased, rinsed, dried, and weighed using an analytical high-precision electronic balance with an accuracy of 0.1 mg at room temperature, in order to obtain the mass loss. Measurements were repeated three times and the average values were obtained.

### 2.5. Tensile Testing

The tensile testing was performed on the electronic tensile testing machine (MTS Systems Corporation, Eden Prairie, MN, USA) at room temperature. The tensile speed was 6 mm/min. The gauge length of 30 mm was set up according to the method used in references [11,18]. The tensile properties of S1, S2, and S3 were measured and recorded.

### 2.6. Fracture Morphology

The fracture morphology of S1, S2, and S3 was examined via a JSM-6360LA scanning electron microscopy (SEM) (JEOL, Tokyo, Japan), respectively. The surface smoothness of the fracture morphology, crack propagation, and dimples were comparatively analyzed.

### 2.7. Statistical Analysis

The data were mean values of three replicated samples. The data were statistically analyzed by using Excel 2010 (Microsoft Corporation, Redmond, WA, USA).

## 3. Results and Discussion

### 3.1. Mass Loss Analysis of Samples Subjected to CE

Table 2 shows the mass loss of original tensile sample (S1), tensile sample without LSP after CE (S2), and tensile sample with LSP after CE (S3), respectively. It can be seen that the average mass of original tensile samples in S1 is 51.63 g. After CE testing, the mass loss of S2 is 0.03 g without LSP, while that of S3 is only 0.01 g with LSP. The mass loss of S2 is thrice as much as that of S3, which indicates that the CE resistance of the material is improved by LSP impacts [2,3]. The high-level compressive residual stresses are generated in the strengthening layer through LSP treatment, and the initiation and growth of cracks during CE can be restrained [12]. The hardness of the material is enhanced [13]. Thus, the sample surface is prevented from being damaged by degradation, leading to the decrease in mass loss of the material. As is known that tensile property is one important index of the material mechanical property, and LSP treatment can also improve the material tensile property after CE theoretically. Based on previous studies [2,3,4], the morphology of surface and cross section was changed during CE, and surface roughness was reduced, micro-hardness was increased, and residual stress was introduced; while the major index, tensile property, has not been involved. In order to study the CE resistance through LSP impacts systematically, effects of LSP on the tensile property of the samples subjected to CE will be specifically researched, and the tensile testing and fracture morphology are to be discussed next.

### 3.2. Analysis of Tensile Property

**Figure 3:**
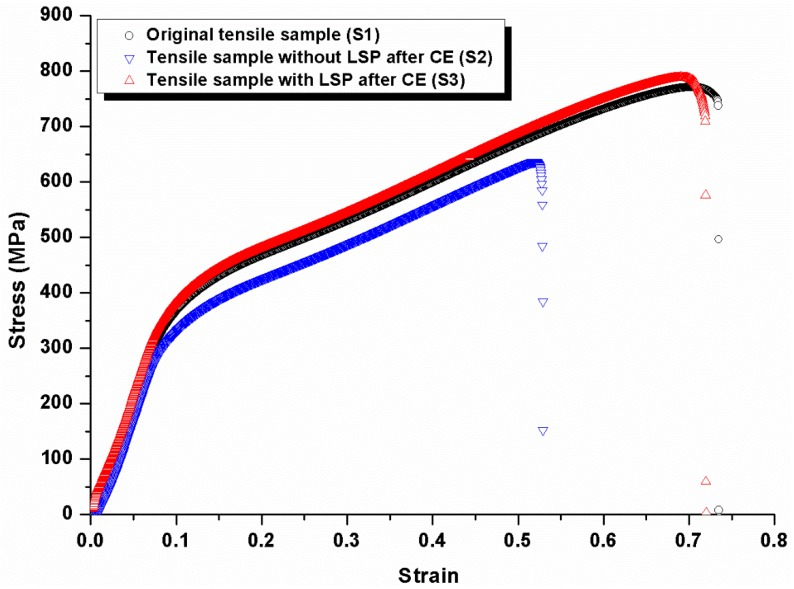
Engineering stress–strain curves of original tensile sample (S1), tensile sample without LSP after CE (S2), and tensile sample with LSP after CE (S3).

Figure 3 gives engineering stress–strain curves of original tensile sample (S1), tensile sample without LSP after CE (S2), and tensile sample with LSP after CE (S3). The tensile samples in the three groups go through the elastic deformation stage and plastic deformation one, finally the fracture happens. There are some differences among the three groups.

#### 3.2.1. Elastic Deformation Stage

During the elastic deformation stage (Figure 3), the relationship between stress and strain is linear relation, and accords with Hooke’s law. Under a certain load, the strain of the material is generated by the applied force. If the force is less than the elastic limit of the material, the induced deformation may be eliminated completely, and the material recovers the original shape. This deformation process is reversible. The elastic limit of S1, S2, and S3 is 364.4 MPa, 333.3 MPa and 383.8 MPa, respectively. It can be deduced that CE destroys the recovery capability of elastic deformation and the life of material is shortened; while LSP can enhance the recovery capability and more applied force is resisted. In the tensile testing, the samples are under uniaxial load and elastic modulus (*E*) is equal to the ratio of the stress to strain. *E* of S1, S2, and S3 are 3968.9, 3869.8, and 4246.0 MPa, respectively. *E* is an important performance parameter of the engineering materials. On the macro level, *E* can evaluate the capability of elastic deformation resistance; on a micro level, it represents bond strength among the atoms, ions and molecules. The factors affecting bond strength will change *E*, such as microstructure, crystalline structure, binding mode, chemical component, and temperature. *E* is regarded as an index to evaluate the ease or complexity of material elastic deformation. The larger the value of *E* is, the greater the applied force to produce elastic deformation is, and the greater the material stiffness is. If the applied force is the same, the larger *E* is, the smaller the elastic deformation is. CE occurs as the collapse of the cavitation bubbles, high temperature, and pressure are induced by micro-streaming during this process. As a result, the material surface is destroyed by stress corrosion due to the corresponding mechanical, chemical, and electrochemical effect [3]. The defect appears in the material structure and the crack is initiated, and the material may fracture even if the force is small. The capability of elastic deformation resistance is reduced and *E* is the smallest. With the help of LSP treatment, the impact load (up to GPa level) will occur on the material surface, high strength impact waves are generated in an ultra-short time (60–100 ns) and propagate within the material. When the pressure of shock waves exceeds the dynamic yield strength, the plastic deformation happens and the high-level compressive residual stress is induced in the strengthening layer. Thus, the mechanical properties of the material will be improved [19], e.g., hardness increases and the surface hardening is produced [20]. The capability of elastic deformation resistance is enhanced and *E* increases, so the CE resistance is improved.

#### 3.2.2. Plastic Deformation Stage

When the stress exceeds the elastic limit of the samples, the plastic deformation stage (Figure 3) is coming, the deformation cannot be recovered completely if the applied force is removed, and a portion of deformation remains. The process of plastic deformation is irreversible. The material of S1, S2, and S3 is polycrystalline composed of many crystalline grains, and the regular space structure of the atoms is formed according to face-centered cubic mode in the grains. However, the atomic structure may have various defects, and the linear jagging of atomic arrangement is dislocation. Because of the dislocation, the atoms are easy to move along the dislocation line when the crystals are subjected to the load, and the capability of deformation resistance is reduced. Hence, the plastic deformation of S1, S2, and S3 is produced. In addition, the slip and twinning are generated during the process of atomic arrangement through the transmitting of dislocation movement, and they are the basic ways of plastic deformation in the grains. The polycrystalline grain boundary is a transition region between two adjacent atoms. The finer the grain is, and the larger the area of grain boundary per unit volume, which is beneficial to the intergranular translational and rotational movements. The grains in S3 are fined due to LSP impacts, and more intergranular translational and rotational movements are induced, so the greatest applied force is needed for plastic deformation. In addition, the elongation and area reduction are main indexes to evaluate the material plasticity. Some metals with ultra-fine grain structure cannot be fractured until elongation up to 300–3000% through the deformation of grain boundary [21]. The corresponding plasticity indexes of S1, S2, and S3 are listed in Table 3. In comparison, the elongation and area reduction of S3 with LSP are a little smaller than that of S1, but larger than that of S2 without LSP. It can be concluded that the tensile property of the samples is improved and the capability of CE resistance is strengthened through LSP treatment. The plastic deformation is induced within the material by LSP impacts on the micro level, which affects the structure and property of the materials [22]. Moreover, the dislocation multiplication is introduced and dislocation density increases. The dislocation intersection happens along different directions, the dislocation movement is hindered and work hardening of the metals is produced. As a result, the hardness, strength, and deformation resistance of the samples are enhanced after LSP treatment [23].

#### 3.2.3. Fracture Stage

The last stage is the fracture (Figure 3), because the plasticity of S1, S2, and S3 is different, the final length and tensile strength are also different as fracture. When the strain is 0.70, S1 fractures with the ultimate tensile strength of 771.5 MPa; when the strain is 0.52, S2 fractures with the ultimate tensile strength of 636.7 MPa; and when the strain is 0.69, S3 fractures with the ultimate tensile strength of 788.9 MPa. LSP enhances the tensile strength of the samples and they may fracture with greater applied force. It is known that high-level compressive residual stresses are generated in the strengthening layer of the material, and the initiation of cracks can be restrained effectively during CE [12]. Thus, the capability of CE resistance is excellent. The tensile property of S1, S2, and S3 is different in Figure 3 due to different treatments, and different fracture morphologies will be obtained according to the matched mechanical property. Therefore, the fracture morphology is compared and analyzed next.

### 3.3. Analysis of Fracture Morphology

The fracture occurs easily at the weakest region of the material and the fracture morphology represents the whole process of the fracture. Lots of fracture problems can be studied through analysis of fracture morphology, including the reason, characteristic, mode, toughness, stress state, and crack propagation rate [14].

#### 3.3.1. Macroscopic Fracture Morphology

**Figure 4:**
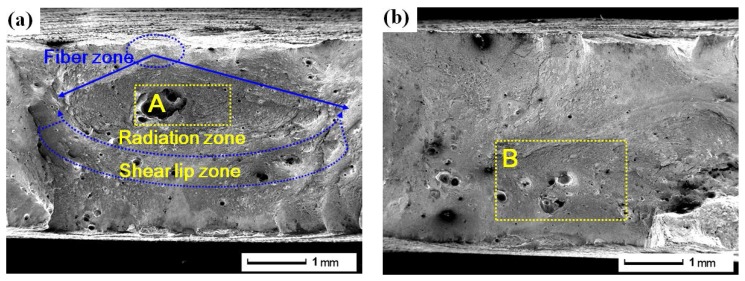

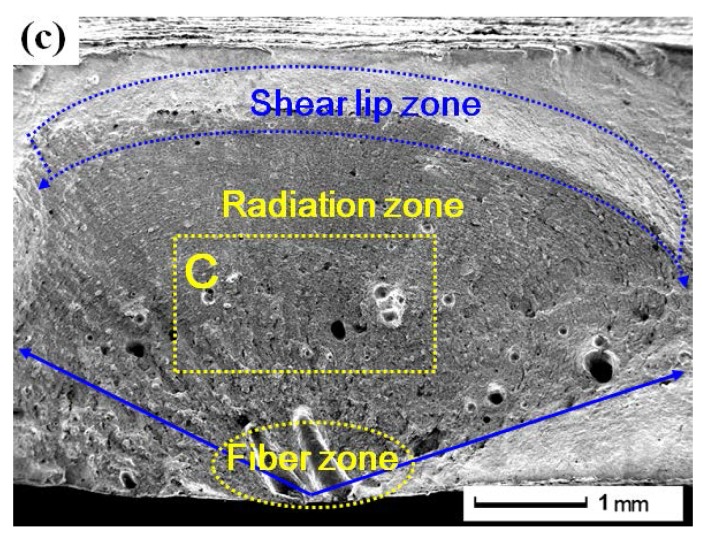
Macroscopic fracture morphology: (**a**) original tensile sample (S1); (**b**) tensile sample without LSP after CE (S2); (**c**) tensile sample with LSP after CE (S3).

Under many circumstances, the fracture characteristic, initiation, and propagation path of the crack can be determined based on the macroscopic fracture morphology [24]. Figure 4 shows the macroscopic fracture morphology of original tensile sample (S1), tensile sample without LSP after CE (S2) and tensile sample with LSP after CE (S3). The fracture morphology of S1 in Figure 4a is similar to that of S3 in Figure 4c, and the fracture surface is composed of fiber zone, radiation zone, and shear lip zone, correspond to crack initiation zone, crack propagation zone, and shear fracture zone, i.e., three elements of fracture. However, the areas of fiber zone, radiation zone, and shear lip zone of S1 in Figure 4a are smaller than that of S3 in Figure 4c. Generally speaking, the area ratio of the three zones to the whole fracture surface is changed with external condition. The plasticity of S3 in Figure 4c is enhanced due to LSP impacts, the deformation process is longer and three zones are larger under the same tensile load. Thus, the tensile property is improved through LSP treatment. In contrast, there are no fiber zone, radiation zone, and shear lip zone in the macroscopic fracture of S2 in Figure 4b. Massive concave–convex structure occurs at the fracture surface, and it indicates that plastic deformation can hardly be generated before abrupt failure, that is, brittle fracture. The brittle fracture is dangerous and occurs abruptly without any harbinger, because the deformation is not obvious enough to be noticed. S2 is destroyed by CE without LSP, corrosion pits appear easily in the welded joint [3], and the crack initiation is formed. The tensile property of S2 is reduced significantly, the fracture occurs under only a little small tensile load, and the plastic deformation stage is short (Section 3.2.2). In Figure 4c, the fiber zone, radiation zone, and shear lip zone of S3 are more obvious than that of S2 in Figure 4b. Although S3 is also subjected to CE and the crack initiation appears at the local region, the tensile property is enhanced owing to LSP treatment and elastoplasticity is enhanced (Section 3.2). Hence, S3 is harder to fracture than S2 under the uniaxial tensile load. Through a longer deformation stage, S3 fractures as no more stress resists the applied force. The deformation can delay damage process of CE, and the sectorial crack propagation region is induced in the microstructure.

#### 3.3.2. High Magnification SEM Micrographs

**Figure 5:**
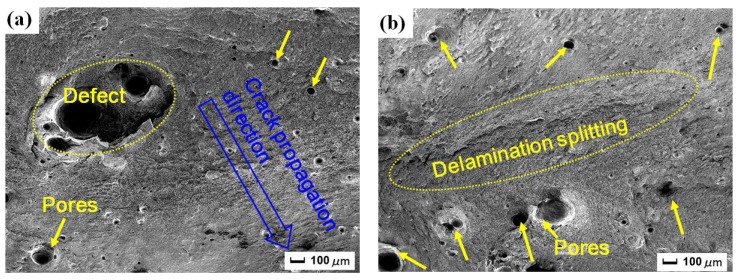

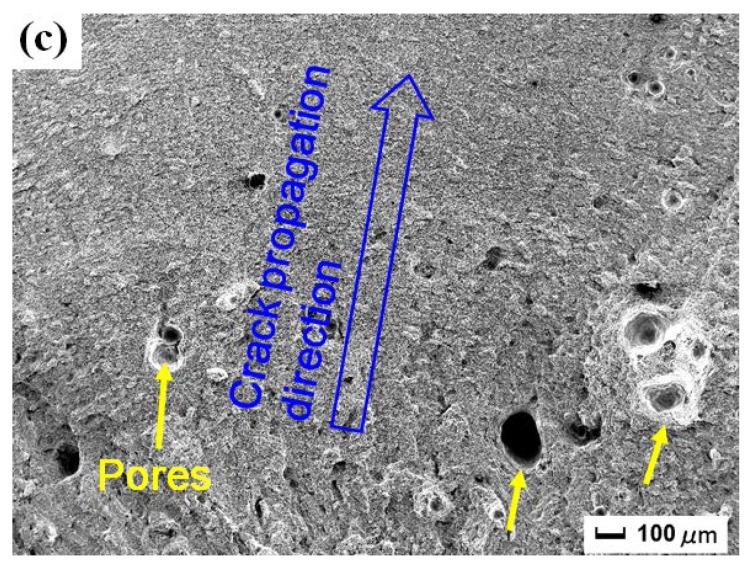
High magnification SEM micrographs of local regions in the yellow square (A), (B), and (C) in Figure 4: (**a**) original tensile sample (S1); (**b**) tensile sample without LSP after CE (S2); (**c**) tensile sample with LSP after CE (S3).

Figure 5 gives high magnification SEM micrographs of fracture morphology in the yellow square (A), (B), and (C) in Figure 4. There are pores of different sizes, but the micromorphology of fracture is different in the three groups. Figure 5a shows the high magnification micrograph in (A) of original tensile sample (S1). The plastic deformation is generated and the direction of crack propagation in the fracture surface is marked in Figure 5a. More pores appear near to the fiber zone. There is a very large pore in Figure 5a, and the pore is the defect of the laser welded sample itself. Because during the laser welding process, some pores are introduced [11], the material defect is formed and becomes crack initiation. The defect and pores grow and their size is to be larger under tensile load. Figure 5b shows the high magnification micrograph in (B) of tensile sample without LSP after CE (S2). It is found that the fracture is irregular with obvious delamination splitting and many pores. The fracture mode of S2 is brittle fracture, so the repetitive stretchable-resilient process does not exist and the delamination splitting is formed by immediate tear. There are lots of pores in the fracture surface of S2 in Figure 5b. More pores occur close to the working face subjected to CE in combination with Figure 4b, more crack initiations are introduced because of CE, and become larger under the tensile load. The material tensile property declines sharply and rapid ultimate fracture happens. Figure 5c shows the high magnification micrograph in (C) of tensile sample with LSP after CE (S3). The direction of crack propagation in the fracture surface is marked in Figure 5c in combination with Figure 4c. The number and size of pores increase closer to the fiber zone, the fiber zone with crack initiation is weak and is the important part. In comparison, the number and size of pores in the fracture of S3 (Figure 5c) is fewer than that of S2 (Figure 5b). It is explained that high-level compressive residual stress induced by LSP can delay or even restrain the growth of nanocracks.

#### 3.3.3. Fracture Morphology in the Fiber Zone

**Figure 6:**
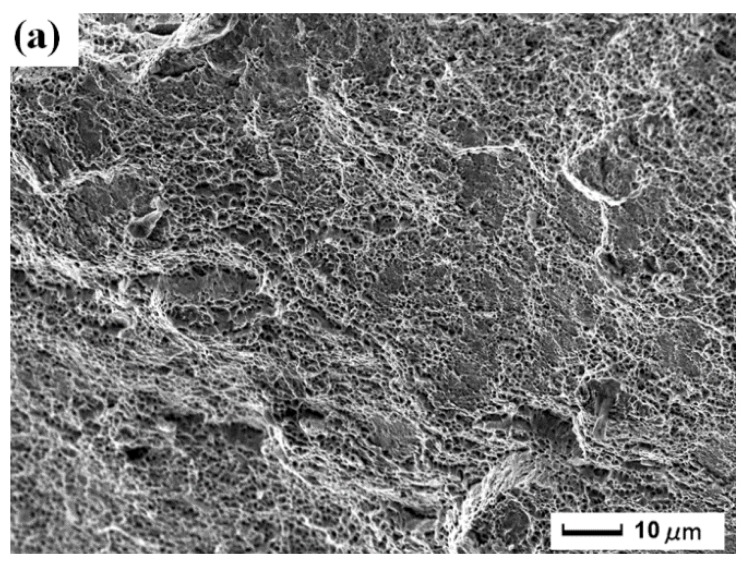

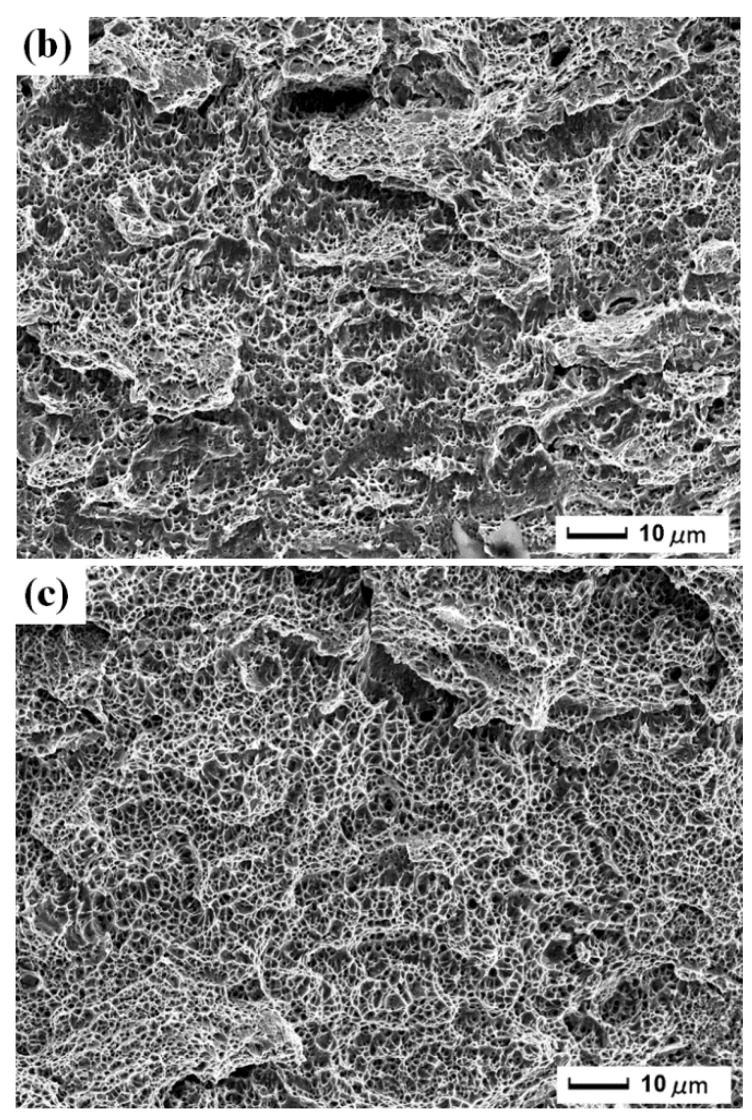
Micrographs of fracture morphology in the fiber zone: (**a**) original tensile sample (S1); (**b**) tensile sample without LSP after CE (S2); (**c**) tensile sample with LSP after CE (S3).

Analysis of fracture micromorphology should be studied near the crack initiation region to reveal the corresponding reason and mechanism, as seen in Figure 6. Figure 6a shows the fracture morphology in the fiber zone of original tensile sample (S1). The crack initiation can hardly appear in S1 and the facture surface is smooth without the damage of CE. Moreover, the quality of the laser weldment itself is excellent [11]. Figure 6b shows the fracture morphology in the fiber zone of tensile sample without LSP after CE (S2). The fracture surface of S2 is coarse and uneven. After CE, nanocracks with different degree of damage appear in local regions within the material. The nanocracks become pores, and grow towards each direction with different sizes. Finally, the ragged fracture surface is formed. Figure 6c shows the fracture morphology in the fiber zone of tensile sample with LSP after CE (S3). The fracture surface of S3 is more smooth and uniform in Figure 6c than that of S2 in Figure 6b. LSP treatment improves the tensile property of the sample (Section 3.2), and restrains the crack initiation. Therefore, the pores are prevented from deteriorating, and the capability of CE resistance is strengthened, which corresponds to the data of CE testing in Section 3.1 and Section 3.2.

#### 3.3.4. Dimples in the Fracture

**Figure 7:**
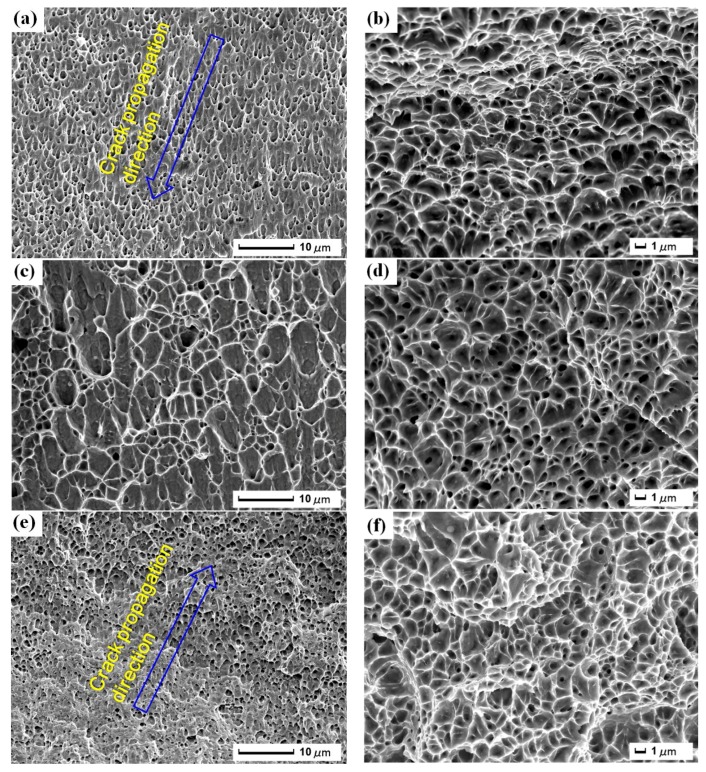
Morphology of the dimples in the fracture surface: (**a**,**b**) original tensile sample (S1); (**c**,**d**) tensile sample without LSP after CE (S2); (**e**,**f**) tensile sample with LSP after CE (S3).

The dimples exist in the fracture in Figure 7, and are generated through the nucleation, growth and interconnection of micro-void. Figure 7a,b show the morphology of the dimples of original tensile sample (S1). Because S1 does not suffer the damage of CE and has the advantage of fiber laser welding [11], the tensile property is good. The distribution of the dimples is uniform with parabolic shape. The micro-voids are subjected to the same stress, and they are stretched along the direction of greater stress, as seen in Figure 7a, which is in accordance with the crack propagation direction. In addition, the fracture with low energy is made under plane strain condition, the size and distribution of the dimples are uniform, and the depth is deep (Figure 7b). The plasticity of the fiber laser weldment is good. Figure 7c,d show the morphology of the dimples of tensile sample without LSP after CE (S2). After CE, various nanocracks appear in S2 and the internal stresses are unbalanced. Then, various dimples are formed under the tensile load, their distribution is non-uniform, and the sample fractures easily. Moreover, the depth of the dimples is shallower in Figure 7c,d. The plasticity of S2 declines because of destruction of CE. Figure 7e,f show the morphology of the dimples of tensile sample with LSP after CE (S3). The dimples are elongated along the unified direction and the elongated direction is in agreement with the crack propagation direction. Their distribution and shape are uniform. The shape of the dimples is closely related to the stress state, and the state is stretch mode. LSP can improve the capability of CE resistance, and the formation and growth of pores are restrained. As a result, the sample is hard to fracture under the same tensile load and the elastic-plastic deformation process is long (Section 3.2). Besides, the dimples are the deepest in Figure 7f and LSP enhances the plasticity of S3.

### 3.4. Strengthening Mechanism Analysis during LSP

**Figure 8:**
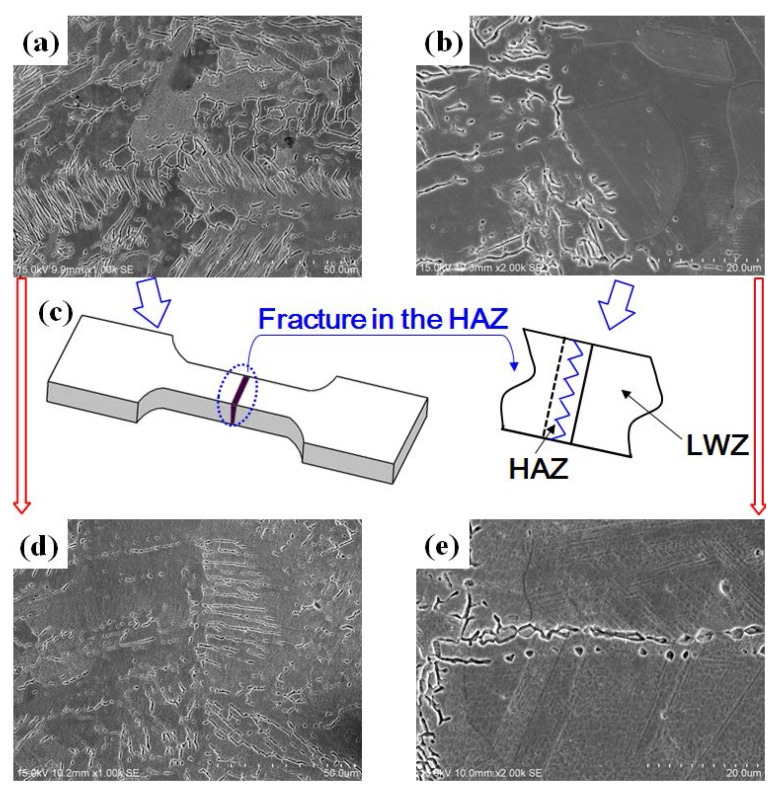
SEM morphology observation of cross sections: (**a**) in the LWZ without LSP [3]; (**b**) in the HAZ without LSP [3]; (**c**) fracture location; (**d**) in the LWZ with LSP [3]; (**e**) in the HAZ with LSP [3].

In order to understand the effects of LSP, the strengthening mechanism is analyzed by the microstructure. The SEM morphology observation of cross sections of ANSI 304 SS laser weldments has been studied [3]. Grains of the columnar ferrite in the LWZ without LSP are coarse in Figure 8a, and the coexistence between the coarse columnar ferrite and austenite exists in the HAZ without LSP in Figure 8b [3]. The ferrite is best hoped to occur, which is beneficial to enhancing mechanical properties of weldments. Thus, the mechanical properties of HAZ are worse than that of LWZ, and the tensile samples fracture in the HAZ, as seen in Figure 8c. Due to LSP, grains of the columnar ferrite are refined in the LWZ and HAZ in Figure 8d,e [3], and grains of austenite are also refined in the HAZ and lots of slip systems occur in grains in Figure 8e, which is beneficial to the improvement of CE resistance by LSP treatment.

**Figure 9:**
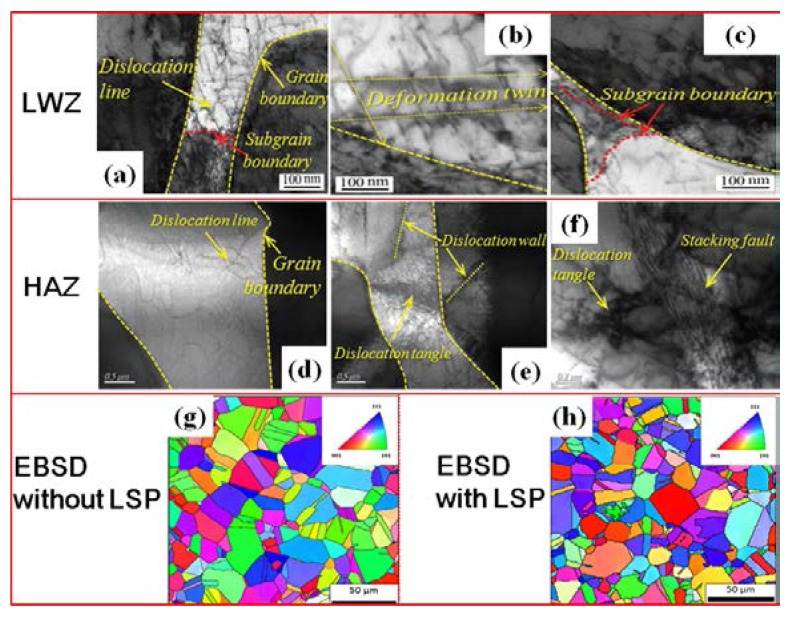
TEM observations and EBSD analysis: (**a**–**c**) in the welding zone with LSP [25]; (**d**–**f**) in the HAZ with LSP [25]; (**g**,**h**) EBSD inverse pole figures without and with LSP [26].

The grain refinement mechanism of stainless steel weldment induced by LSP is also verified by transmission electron microscopy (TEM) observations [25] and electron backscattered diffraction (EBSD) analysis [26]. The microstructures in the welding zone and HAZ are affected by LSP, and the refined grains are generated in view of dislocation movement in Figure 9a–f [25]. The formation of dislocation tangles and dislocation walls occur due to the pile up of dislocation lines. Subgrain boundaries are formed, developed, and evolved into new grain boundaries by TEM observations [25]. As a result, the coarse grain of original stainless steel with the size of more than 30 μm can be changed into the refined grains with the size of 15 μm, and the distribution is more homogeneous and uniform after LSP by EBSD analysis in Figure 9g,h [26]. Thus, for S3 with LSP, the tensile property can be improved and the corresponding CE resistance is strengthened at the macro level.

## 4. Conclusions

In this study, the effects of laser shock processing (LSP) on the tensile properties of the laser weldments subjected to cavitation erosion (CE) were studied, and the corresponding experimental data and tensile fractures were measured and analyzed (*n* = 3). It was found that LSP can improve the capability of CE resistance. Some conclusions obtained from this investigation were listed below:

(1) The mass loss of tensile sample without LSP after CE (S2) was thrice as much as that of tensile sample with LSP after CE (S3).
(2) During the elastic deformation stage, the elastic limit of original tensile sample (S1), S2, and S3 was 364.4, 333.3, and 383.8 MPa, respectively. The elastic modulus of S1, S2, and S3 were 3968.9, 3869.8, and 4246.0 MPa, respectively. During the plastic deformation stage, the elongation and area reduction of S3 with LSP were a little smaller than that of S1, but larger than that of S2 without LSP. During the fracture stage, S1, S2, and S3 fractured with the ultimate tensile strength of 771.5, 636.7, and 788.9 MPa, respectively.
(3) The fiber zone, radiation zone, and shear lip zone of S3 were more obvious than that of S1 and S2. The fracture mode of S2 was brittle fracture. The number and size of pores in the fracture surface of S3 were the smallest. The fracture surface of S3 was smoothest and the most uniform. The dimples of S3 were elongated along the unified direction and the elongated direction was in agreement with the crack propagation direction. Their distribution and shape were uniform. The dimples were the deepest and LSP enhanced the plasticity of S3.

## Figures and Tables

**Table 1:** Testing parameters in cavitation erosion tests

Output Power (W)	Frequency (kHz)	Diameter of Vibrating Horn (mm)	Pulsed Mode			Total Testing Time (h)	Temperature (°C)
			On-Time (s)	Off-Time (s)			
840	20	20	1.5		3	6	20

**Table 2:** Mass loss of original tensile sample (S1), tensile sample without LSP after CE (S2), and tensile sample with LSP after CE (S3)

Group	Sample	Without LSP Impacts (S2)	With LSP Impacts (S3)
Mass before CE (g)	First sample	51.633	51.631
	Second sample	51.628	51.622
	Third sample	51.636	51.630
	Mean values	51.63	51.63
Mass after CE (g)	First sample	51.598	51.624
	Second sample	51.603	51.614
	Third sample	51.611	51.625
	Mean values	51.60	51.62
Mass loss (g)	First sample	0.035	0.007
	Second sample	0.025	0.008
	Third sample	0.025	0.005
	Mean values	0.03	0.01

**Table 3:** Elongation and area reduction of original tensile sample (S1), tensile sample without LSP after CE (S2), and tensile sample with LSP after CE (S3)

Tensile Property	Sample	Original Tensile Sample (S1)	Tensile Sample without LSP after CE (S2)	Tensile Sample with LSP after CE (S3)
Elongation	First sample	0.708	0.523	0.689
	Second sample	0.702	0.522	0.691
	Third sample	0.690	0.529	0.699
	Mean values	0.70	0.52	0.69
Area reduction	First sample	0.411	0.282	0.340
	Second sample	0.412	0.281	0.342
	Third sample	0.416	0.288	0.343
	Mean values	0.41	0.28	0.34

## References

[1] Sreedhar B.K., Albert S.K., Pandit A.B. (2017). Cavitation damage: Theory and measurements—A review. Wear.

[2] Zhang L., Lu J.Z., Zhang Y.K., Ma H.L., Luo K.Y., Dai F.Z. (2017). Effects of laser shock processing on morphologies and mechanical properties of ANSI 304 stainless steel weldments subjected to cavitation erosion. Materials.

[3] Zhang L., Zhang Y.K., Lu J.Z., Dai F.Z., Feng A.X., Luo K.Y., Zhong J.S., Wang Q.W., Luo M., Qi H. (2013). Effects of laser shock processing on electrochemical corrosion resistance of ANSI 304 stainless steel weldments after cavitation erosion. Corros. Sci..

[4] Zhang L., Luo K.Y., Lu J.Z., Zhang Y.K., Feng A.X. (2013). Effects of laser shock processing on cavitation erosion resistance of laser weldments. Chin. J. Lasers.

[5] Han M.X., Liu Y.S., Wu D.F., Zhao X.F., Tan H.J. (2017). A numerical investigation in characteristics of flow force under cavitation state inside the water hydraulic poppet valves. Int. J. Heat Mass Transf..

[6] Kang C., Mao N., Zhang W.B., Gu Y.P. (2017). The influence of blade configuration on cavitation performance of a condensate pump. Ann. Nucl. Eng..

[7] Kwok C.T., Man H.C., Cheng F.T., Lo K.H. (2016). Developments in laser-based surface engineering processes: With particular reference to protection against cavitation erosion. Surf. Coat. Technol..

[8] Gujba A.K., Medraj M. (2014). Laser peening process and its impact on materials properties in comparison with shot peening and ultrasonic impact peening. Materials.

[9] Luo S.H., Zhou L.C., Wang X.D., Cao X., Nie X.F., He W.F. (2018). Surface nanocrystallization and amorphization of dual-phase TC11 titanium alloys under laser induced ultrahigh strain-rate plastic deformation. Materials.

[10] Li X.Y., Yan Y.G., Ma L., Xu Z.M., Li J.G. (2004). Cavitation erosion and corrosion behavior of copper-manganese-aluminum alloy weldment. Mater. Sci. Eng. A.

[11] Zhang L., Lu J.Z., Luo K.Y., Feng A.X., Dai F.Z., Zhong J.S., Luo M., Zhang Y.K. (2013). Residual stress, micro-hardness and tensile properties of ANSI 304 stainless steel thick sheet by fiber laser welding. Mater. Sci. Eng. A.

[12] Chandrasekar G., Kailasanathan C., Verma D.K. (2017). Investigation on un-peened and laser shock peened weldment of Inconel 600 fabricated by ATIG welding process. Mater. Sci. Eng. A.

[13] Lu G.X., Liu J.D., Qiao H.C., Zhou Y.Z., Jin T., Zhao J.B., Sun X.F., Hu Z.Q. (2017). Effect of laser shock on tensile deformation behavior of a single crystal nickel-base superalloy. Mater. Sci. Eng. A.

[14] Zhang L., Luo K.Y., Lu J.Z., Zhang Y.K., Dai F.Z., Zhong J.W. (2011). Effects of laser shock processing with different shocked paths on mechanical properties of laser welded ANSI 304 stainless steel joint. Mater. Sci. Eng. A.

[15] Chen W.G., Gu C.Q., Shen F.S. (1998). Correlation of cavitation erosion resistance with mechanical properties of steels. J. Hydroelectr. Eng..

[16] Xiong J.Z., Yang X.Q., Lin W., Liu K.X. (2018). Evaluation of inhomogeneity in tensile strength and fracture toughness of underwater wet friction taper plug welded joints for low-alloy pipeline steels. J. Manuf. Processes.

[17] (2009). Standard Test Method for Cavitation Erosion Using Vibratory Apparatus.

[18] Zhang Y.K., Zhang L., Luo K.Y., Sun G.F., Lu J.Z., Dai F.Z., Zhong J.W. (2012). Effects of laser shock processing on mechanical properties of laser welded ANSI 304 stainless steel joint. Chin. J. Mech. Eng..

[19] Xiong Y., He T.T., Guo Z.Q., He H.Y., Ren F.Z., Volinsky A.A. (2013). Effects of laser shock processing on surface microstructure and mechanical properties of ultrafine-grained high carbon steel. Mater. Sci. Eng. A.

[20] Lu J.Z., Wu L.J., Sun G.F., Luo K.Y., Zhang Y.K., Cai J., Cui C.Y., Luo X.M. (2017). Microstructural response and grain refinement mechanism of commercially pure titanium subjected to multiple laser shock peening impacts. Acta Mater..

[21] Chen L.F., Xiong Y., Li H.P., Lu Y., Ren F.Z. (2016). Microstructure evolution and mechanical properties of 316LN austenitic stainless steel after tensile deformation at different temperatures. Trans. Mater. Heat Treat..

[22] Moćko W., Radziejewska J., Sarzyński A., Strzelec M., Marczak J. (2017). Analysis of the plastic deformation of AISI 304 steel induced by the nanosecond laser pulse. Opt. Laser Technol..

[23] Agrawal A.K., Singh A. (2017). Limitations on the hardness increase in 316L stainless steel under dynamic plastic deformation. Mater. Sci. Eng. A.

[24] Paredes M., Wierzbicki T., Zelenak P. (2016). Prediction of crack initiation and propagation in X70 pipeline steels. Eng. Fract. Mech..

[25] Lu J.Z., Zhang W.Q., Jing X., Wu L.J., Luo K.Y. (2017). Microstructural evolution in the welding zone of laser shock peened 316L stainless steel tube. J. Laser Appl..

[26] Lu J.Z., Deng W.W., Luo K.Y., Wu L.J., Lu H.F. (2017). Surface EBSD analysis and strengthening mechanism of AISI304 stainless steel subjected to massive LSP treatment with different pulse energies. Mater. Charact..

